# Estradiol Mediates Astrocyte-Neuron Communication in the Hippocampus

**DOI:** 10.1007/s12035-025-04905-6

**Published:** 2025-04-10

**Authors:** Julianna Goenaga, Carmen Nanclares, Megan Hall, Paulo Kofuji, Paul G. Mermelstein, Alfonso Araque

**Affiliations:** Department of Neuroscience, University of Minnesota, 6 - 145 Jackson Hall, 321 Church Street SE, Minneapolis, MN 55455 USA

**Keywords:** Astrocytes, Estrogen, Hippocampus, Calcium, Slow Inward Currents

## Abstract

Accumulating evidence has revealed the existence of functional astrocyte-neuron communication based on the ability of astrocytes to respond to neurotransmitters and release gliotransmitters. However, little is known about how other signaling molecules, such as hormones, impact astrocyte function. Estradiol (E2) is an important hormone known to regulate neuronal activity, synaptic transmission, plasticity, and animal behavior. However, whether E2 specifically signals to astrocytes in situ and the functional consequences on astrocyte-neuron communication remain unknown. Therefore, we investigated the impact of estradiol on astrocyte activity and astrocyte-neuron communication in the mouse hippocampus. Using an RNAscope approach, we determined that estrogen receptors (ERα and ERβ) are expressed in astrocytes in both female and male mice. In both sexes, confocal imaging of hippocampal slices determined that astrocytes respond to locally applied E2 with calcium elevations. In pyramidal neurons, slow inward currents (SICs) are mediated by the activation of extrasynaptic NMDA receptors and indicate gliotransmission. Electrophysiological recordings of hippocampal neurons determined that E2 increases the frequency, but not the amplitude, of SICs. We also recorded excitatory synaptic transmission evoked by Schaffer collateral stimulation. Here, only in females, did E2 produce a reduction in excitatory synaptic transmission. The E2-induced effects on the astrocyte calcium signal and gliotransmission were prevented by the broad estrogen receptor antagonist ICI 182,780. Taken together, these results demonstrate the existence of estradiol-mediated astrocyte-neuron communication in both female and male mice. They reveal that E2 can signal to astrocytes and, through this signaling, E2 may regulate neuronal activity and synaptic transmission.

## Introduction

Estrogens are a class of steroid hormones that have multiple actions throughout the body with important health implications. Classically, estrogens bind to the nuclear estrogen receptors alpha and/or beta (ERα and ERβ), which act as transcription factors leading to changes in gene expression and protein synthesis, ultimately leading to alterations in behavior [1]. Estrogens also act through membrane-localized estrogen receptors, leading to the regulation of intracellular signaling cascades and rapid alterations in cell excitability [1–4]. Membrane-bound estrogen receptors have been found in various brain regions, including the hypothalamus, nucleus accumbens, and hippocampus [4–9]. Estradiol (E2), the principal estrogen, has been shown to regulate neuronal calcium dynamics [1, 10], neuronal excitability [11], and synaptic transmission [12–14] through, at least in part, “fast” actions through membrane receptors.

Most research examining rapid estrogen signaling has focused on neuronal cells, and thus little is known about the impact of E2 on astrocytes. Several studies have shown the existence of estrogen receptors in cultured astrocytes from the cortex and hypothalamus [5–7, 15–20]. However, evidence supporting the expression of estrogen receptors (ERs) in astrocytes in situ is limited [21–25].

Astrocytes were thought to play a passive role in the brain, such as maintaining the blood–brain barrier and providing metabolic support [26]. However, accumulating evidence shows that astrocytes play a pivotal and active role in neuronal communication leading to the conceptual framework of the tripartite synapse [26–28]. One way that astrocytes interact with neurons is by releasing gliotransmitters in response to neurotransmitters or other gliotransmitters [28–36]. However, whether other signaling molecules, such as hormones, affect astrocyte physiology and astrocyte-neuron interaction remains unknown.

Using hippocampal slices, we have investigated the calcium responsiveness of hippocampal astrocytes to local application of E2, and whether E2 signaling in astrocytes stimulates gliotransmission and regulates synaptic transmission. Our findings show that astrocytes respond to E2 with transient intracellular calcium elevations. Associated with this astrocyte calcium signal, we observed an increase in the frequency of extrasynaptic NMDAR-mediated slow inward currents (SICs) in neurons, which are known to be mediated by the release of the gliotransmitter glutamate by astrocytes [37–40]. Furthermore, E2 induced a transient depression of Schaffer collateral-evoked excitatory synaptic transmission in female but not male mice. This E2-induced synaptic regulation observed in female mice was sensitive to the broad estrogen receptor antagonist ICI 182,780 (ICI) and was absent in conditional IP3R2^−/−^ mice, which largely lack calcium signaling in astrocytes [41–44]. It was also abolished by antagonizing type 1 adenosine receptors (A1Rs). Taken together, these results indicate that E2 rapidly signals to astrocytes through estrogen receptor activation, regulates their intracellular calcium dynamics through calcium mobilization, and stimulates the gliotransmitter release of glutamate that modulates neuronal excitability. Notably, while E2-evoked astrocyte calcium signal and glutamatergic gliotransmission similarly occurred in both female and male mice, the effects of E2 on synaptic transmission were only observed in female mice.

## Results

### Hippocampal Astrocytes Express Estrogen Receptors

**Fig. 1:**
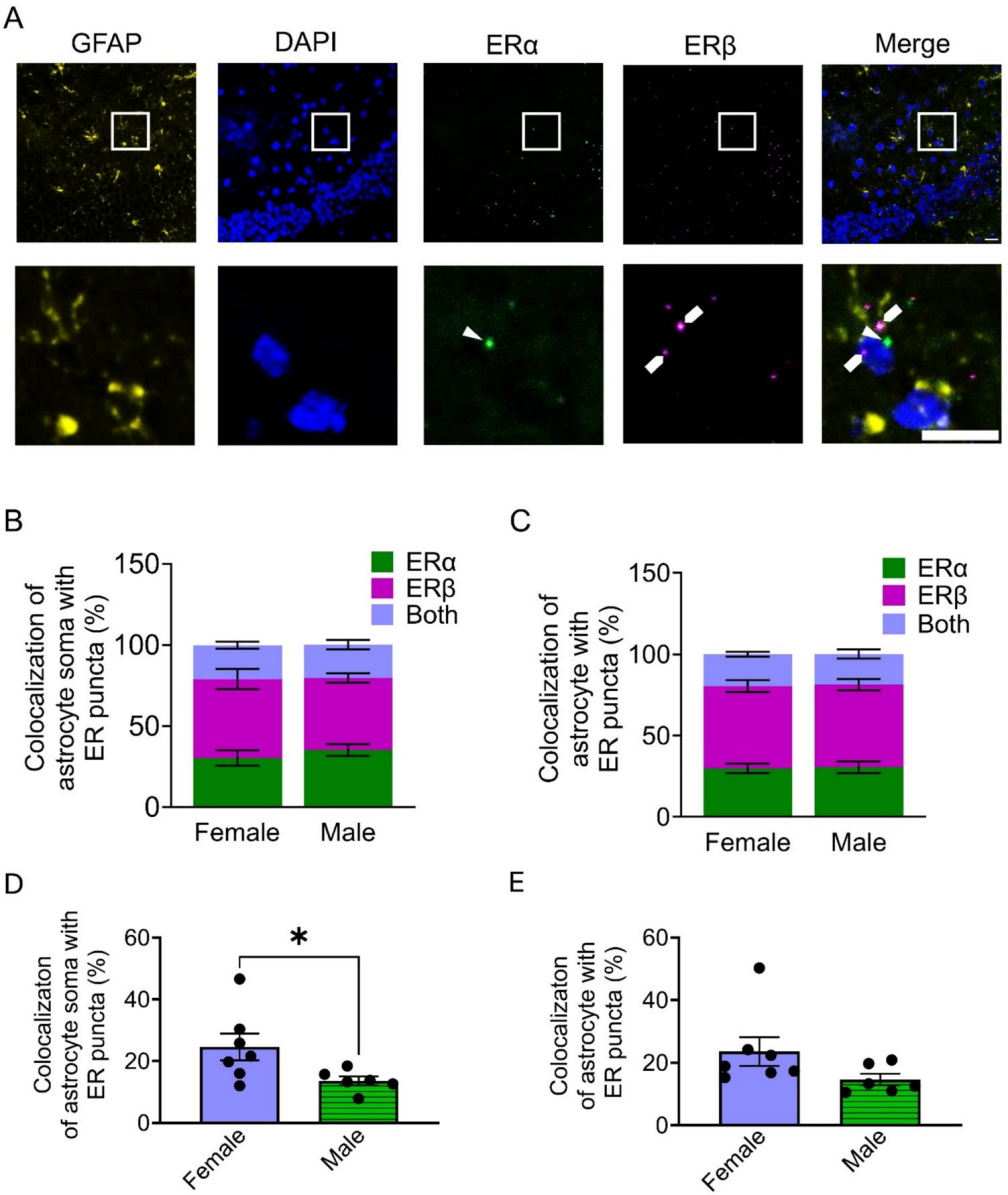
Expression of estrogen receptor subtypes in astrocytes among female and male mice. (**A**) Representative images of expression of GFAP, DAPI, ERα, ERβ, and merge of all four images (scale bar = 50 µm). **Inset:** zoom in image. Colocalization indicated by arrowhead (scale bar = 20 µm). (**B**) Breakdown of ER subtype expression in astrocyte somas in female and male mice. (**C**) Breakdown of ER subtype expression colocalized with GFAP in female and male mice. (**D**) Percentage of colocalized ER puncta in astrocyte somas (GFAP + DAPI) for female (24.6 ± 4.3%) and male mice (13.6 ± 1.4%). Two-tailed Student’s unpaired t-test. (**E**) Percentage of colocalized ER puncta with GFAP for female (23.59 ± 4.6%) and male mice (14.6 ± 1.8%). Female (n = 7 mice) and male (n = 6 mice). Mann–Whitney U test. Data are expressed as mean ± standard error of the mean (SEM), * *p* < 0.05

To investigate the involvement of astrocytes in E2 signaling within the hippocampus, we first examined the expression of estrogen receptors (ER) by astrocytes in the CA1 region of the hippocampus using immunohistochemistry and RNAscope. We simultaneously visualized astrocytes (immunohistochemically identified with the specific astrocytic protein GFAP), cells (using DAPI as the fluorescent stain of nuclei), and mRNAs of ERα and ERβ using the RNAscope assay (Fig. 1A). Astrocyte somas were identified by colocalization of both DAPI and GFAP immunofluorescence. We then quantified the colocalization of astrocytic somas with ERα and ERβ puncta. We found that ERα was similarly expressed in astrocytes of both female (30.2 ± 4.7%) and male (35.2 ± 3.7%) mice (Fig. 1B). Likewise, ERβ was also similarly expressed in astrocytes of female and male mice (48.7 ± 6.2% and 44.4 ± 2.8%, respectively) (Fig. 1B). Moreover, simultaneous expression of both ERs was observed in 20.8 ± 2.1% of astrocytic somas in females and 20.5 ± 3.0% in males (Fig. 1B). We further examined the ER expression in both the soma and processes of astrocytes, quantifying the colocalization of GFAP with ERα and ERβ puncta. Colocalization of ERα and GFAP was observed in 29.8 ± 3.0% in females and 30.6 ± 3.6% in males (Fig. 1C). Colocalization of ERβ and GFAP was more predominant, as it occurred in 50.6 ± 3.6% in females and 50.8 ± 3.5% in males) (Fig. 1C). Co-expression of both ERs was observed in 19.6 ± 1.5% of astrocytes in females and 18.8 ± 2.7% of astrocytes in males (Fig. 1C). Thus, estrogen receptors ERα and ERβ are expressed in hippocampal astrocytes of both males and females, with a prevalence of ERβ expression in both sexes. However, astrocytic somas of female mice express overall more ER than males (24.6 ± 4.3% in females; 13.6 ± 1.4% in males) (Fig. 1D). This sex difference in the expression of ER was not observed when considering both the astrocytic somas and processes (23.6 ± 4.6% in females; 14.6 ± 1.8% in males) (Fig. 1E). Taken together, these data indicate that astrocytes express ER in the hippocampus, with an increase in the overall expression in female mice when compared to males.

### E2 Generates Intracellular Calcium Elevation on Astrocytes

**Fig. 2:**
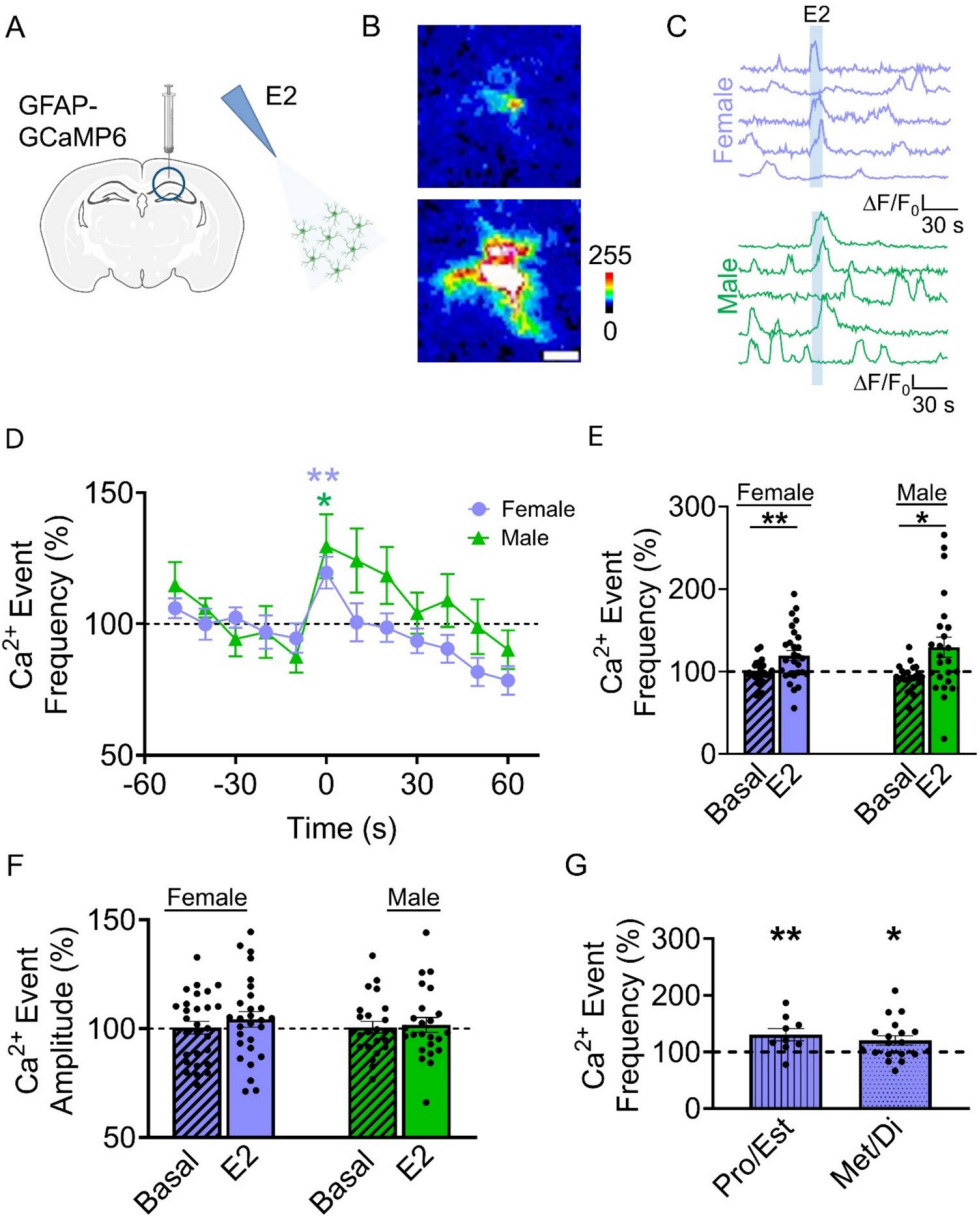
E2 evokes calcium elevation in astrocytes. (**A**) Scheme of experimental approach. **Left:** GFAP-GCaMP6 viral injection into the CA1 region of the hippocampus. **Right:** Estradiol (10 µM) puff. (**B**) Pseudo-color images showing changes in fluorescence in GCaMP6 expressing astrocytes during baseline (top) and E2 (bottom) application (scale bar: 20 µm). (**C**) Representative traces of Ca^2+^ fluorescence intensity in female (light purple; scale bar: 1 ∆F/F_0_, 30 s) and male (green; scale bar: 1 ∆F/F_0_, 30 s) mice in response to E2 application (light blue shading). (**D**) Normalized Ca.^2+^ event frequency per 10 s with E2 applied at 0 s (Females: n = 28 experimental planes of view from n = 10 mice; Males: n = 24 experimental planes of view from n = 7 mice). (**E**) Normalized calcium events relative to baseline during E2 application (Basal: 98 ± 2.7%, E2: 119.5 ± 6.1% for females; Basal: 93 ± 3%, E2: 129.6 ± 12.2% for males). (**F**) Normalized mean calcium event amplitude relative to baseline during E2 application (Basal: 100.5 ± 2.9%, E2: 104.3 ± 3.6% for females; Basal: 100.6 ± 2.7%, E2: 101.7 ± 3.4% for males). (**G**) Normalized calcium events relative to baseline during E2 application in proestrus/estrus (130.5 ± 10.8%) and metestrus/diestrus (120.3 ± 8.2%) (pro/est: n = 9 experimental planes of view from n = 5 mice; met/di: n = 19 experimental planes of view from n = 5 mice). Two-tailed Student’s paired t-test. Data are expressed as mean ± standard error of the mean (SEM). * *p* < 0.05, ** *p* < 0.005

The excitability of astrocytes, unlike neurons, is encoded by variations in cytosolic calcium levels [45]. To investigate whether hippocampal astrocytes express functional ER, we recorded the intracellular Ca^2+^ levels of astrocytes located in the CA1 region of the hippocampus and analyzed the responses of the astrocyte somas to local application of E2. To monitor astrocyte calcium activity, female and male mice received stereotaxic injections of AAV5-GfapABC1D-cytoGCaMP6f, to express the calcium indicator GCaMP6f under the astrocytic promoter GFAP (Fig. 2A). This method was shown to specifically express GCaMP6f in astrocytes across multiple brain areas, including the hippocampus [34–36, 46–49]. Local application of E2 increased the frequency of Ca^2+^ events in both female and male mice (Fig. 2B-E). E2 did not increase Ca^2+^ events amplitude for either female or male mice (Fig. 2F). Taken together, this indicates that astrocytes respond to E2 signaling with increases in their calcium activity.

ER levels are known to change depending on the female estrus cycle [50]. To determine if the astrocytic response to E2 would vary depending on this factor, we studied the Ca^2+^ levels of astrocytes in different phases of the estrus cycle of female mice. Mice were swabbed post-mortem, and cells were analyzed using light microscopy. Proestrus and estrus phases were compared to metestrus and diestrus phases. Interestingly, baseline astrocyte calcium activity was higher (*p* < 0.05, Kruskal–Wallis) in slices obtained from females in proestrus/estrus (3.7 ± 0.5/10 s (mean event frequency)) in comparison to females in metestrus/diestrus (2.2 ± 0.6) or males (1.5 ± 0.2). Since baseline calcium activity fluctuated across the estrous cycle, group comparisons following E2 exposure were performed using a normalized baseline. Regardless of the estrous phase, E2 similarly increased the Ca^2+^ event frequency in astrocytes (Fig. 2G), indicating that astrocytes respond to E2 across all stages of the estrous cycle.

**Fig. 3:**
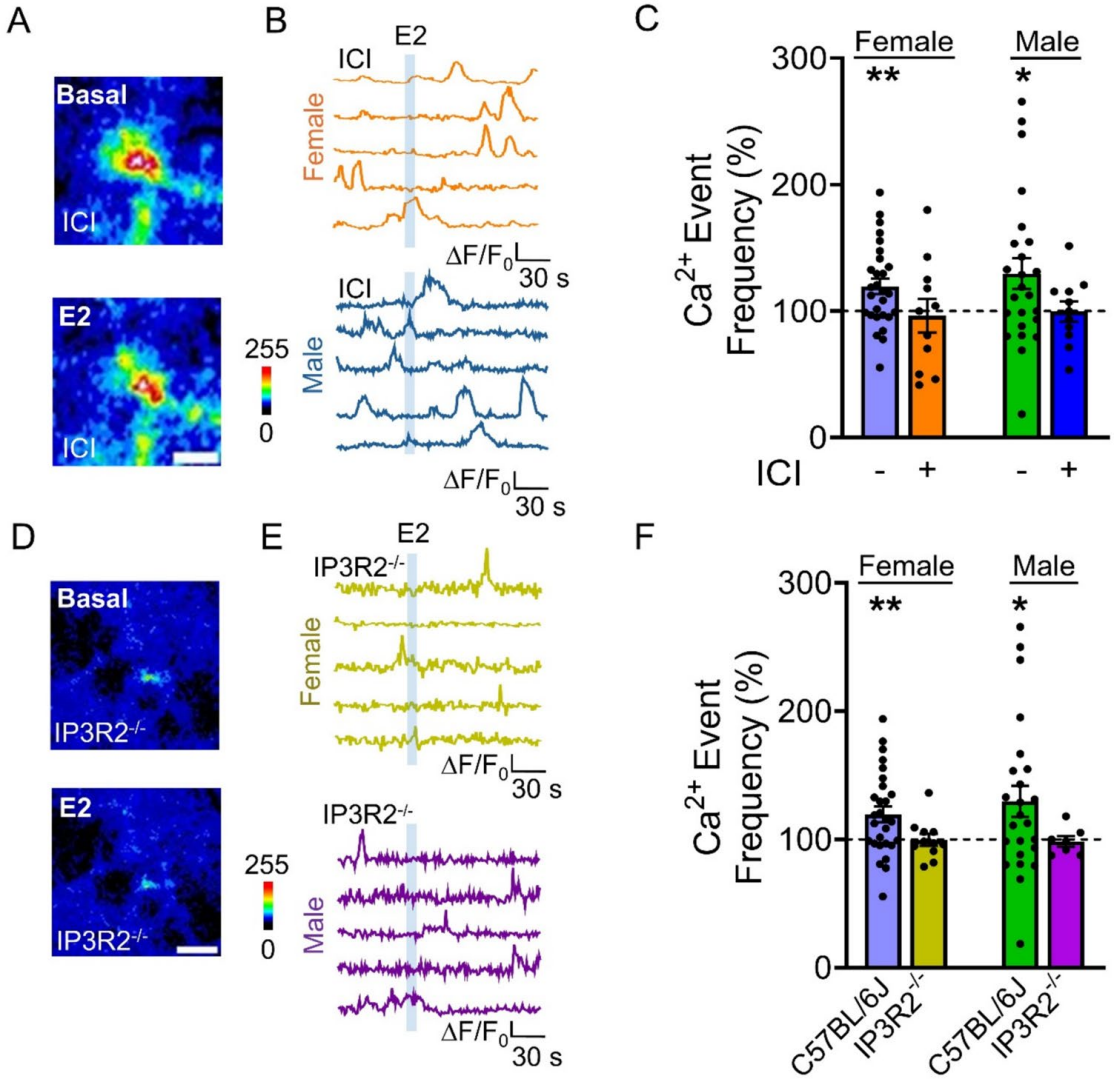
E2 effects are mediated by estrogen receptors and IP3 signaling. (**A**) Pseudo color images showing changes in fluorescence in GCaMP6 expressing astrocytes during baseline and E2 (10 µM) application (scale bar: 20 µm) in the presence of ICI 182,780 (ICI; 1 µM). (**B**) Representative traces of Ca^2+^ fluorescence intensity in female (orange; scale bar: 1 ∆F/F_0_, 30 s) and male (blue; scale bar: 0.5 ∆F/F_0_, 30 s) mice in response to E2 application (light blue shading) in the presence of ICI. (**C**) Normalized calcium events relative to baseline during E2 application in the absence (119.5 ± 6.1%) or presence (96.3 ± 13.2%) of ICI for females and in the absence (129.6 ± 12.2%) or presence (99.8 ± 8.1%) of ICI for males. Females ICI: n = 11 experimental planes of view from n = 5 mice; Males ICI: n = 11 experimental planes of view from n = 4 mice. (**D**) Pseudo color images showing changes in fluorescence in GCaMP6 expressing astrocytes from IP3R2^−/−^ mice during baseline and E2 (10 µM) application (scale bar: 20 µm). (**E**) Representative traces of Ca^2+^ fluorescence intensity in IP3R2^−/−^ female (yellow; scale bar: 1 ∆F/F_0_, 30 s) and IP3R2^−/−^ male (purple; scale bar: 1 ∆F/F_0_, 30 s) mice in response to E2 application (light blue shading). **F)** Normalized calcium events relative to baseline during E2 application in female C57BL/6 J (119.5 ± 6.1%) and IP3R2^−/−^ (99.6 ± 4.7%) mice and in male C57BL/6 J (129.6 ± 12.2%) and IP3R2^−/−^ (98.4 ± 4.1%) mice. Females IP3R2^−/−^: n = 11 experimental planes of view from n = 2 mice; Males IP3R2.^−/−^: n = 7 experimental planes of view from n = 3 mice. Two-tailed Student’s paired t-test. Data are expressed as mean ± standard error of the mean (SEM), * *p* < 0.05, ** *p* < 0.005

We then asked whether the E2-induced astrocyte calcium signal was mediated by estrogen receptor activation. We tested the effects of the broad antagonist of estrogen receptors ICI on the astrocyte calcium responsiveness to E2. We found that the increase in the Ca^2+^ event frequency evoked by E2 in control conditions was prevented in the presence of ICI 182,780 (Fig. 3A-C), indicating that the E2-evoked Ca^2+^ elevations observed in control were mediated by estrogen receptor activation. Finally, we explored the intracellular signaling cascade that governs the astrocyte response to E2. One of the primary pathways for calcium signaling in astrocytes involves IP3 receptor-mediated calcium mobilization from the endoplasmic reticulum [33, 45, 51]. To investigate the role of IP3 signaling, we utilized IP3R2^−/−^ mice that exhibit impaired G protein-coupled receptor-mediated astrocyte calcium signaling [44, 52, 53]. No significant changes in calcium signaling were observed in IP3R2^−/−^ mice following exposure to E2 (Fig. 3D-F), indicating that E2-induced increases in astrocyte calcium are mediated by IP3 receptor-mediated signaling in astrocytes.

### E2 Stimulates the Release of the Gliotransmitter Glutamate from Astrocytes

**Fig. 4:**
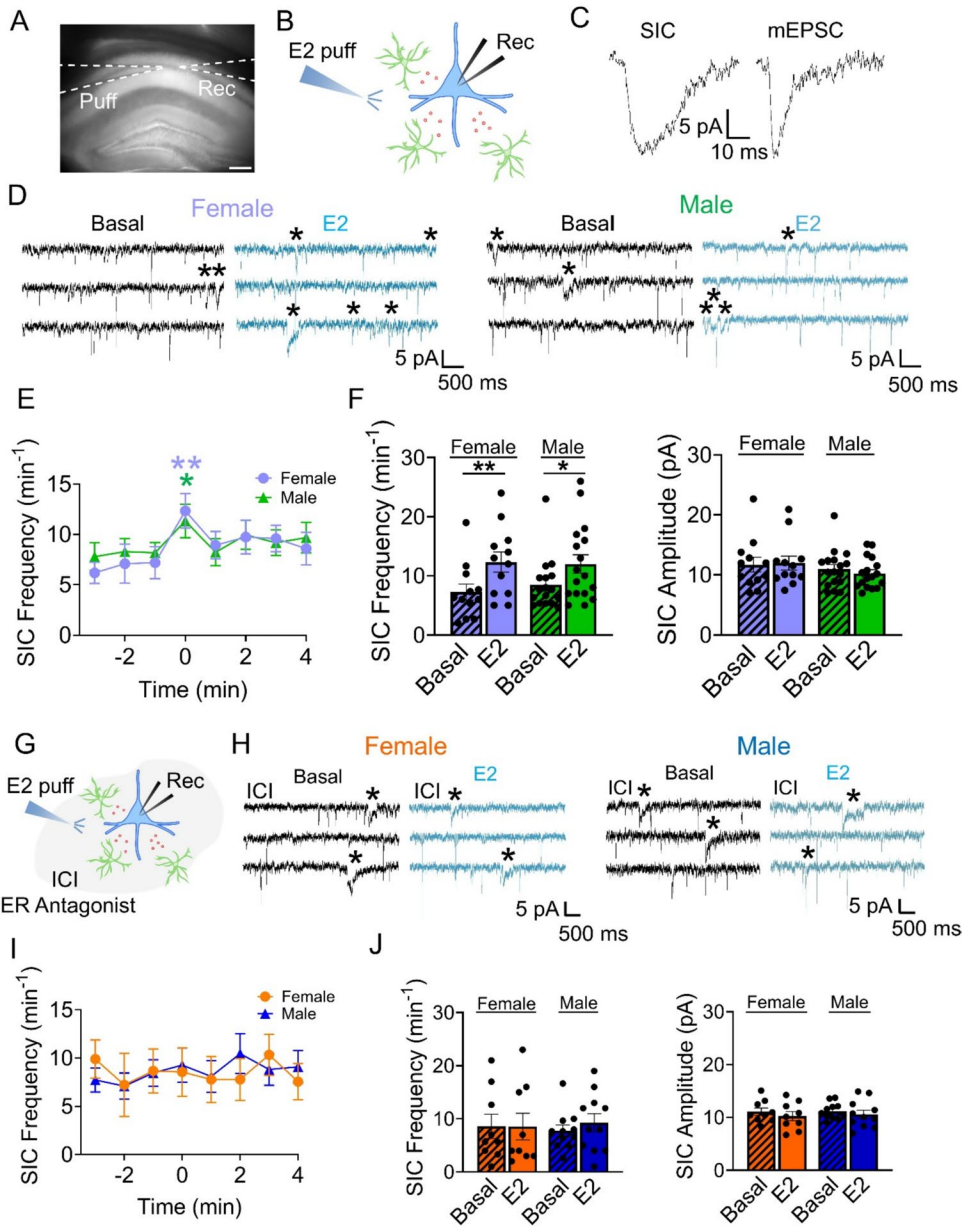
E2 increases astrocyte glutamate release. (**A**) Differential interference contrast (DIC) image of a hippocampal brain slice. Puff: puffing/pressure pulse pipette; Rec: recording pipette (scale bar: 250 µm). (**B**) Scheme of the experimental approach: a recording pipette was placed in a hippocampal pyramidal neuron (blue) and a puffing pipette was located nearby. Astrocytes are depicted in green and glutamate in red. (**C**) Representative traces of a slow inward current (SIC) and a miniature excitatory postsynaptic current (mEPSC). Note the slower kinetics of the SIC. (**D**) **Left:** Representative SIC traces before and after application of E2 (10 µM) in female mice. Note the increase in SICs (asterisks) after the E2 puff (scale bar: 5 pA, 500 ms). **Right:** Representative SIC traces before and after application of E2 in male mice. Note the increase in SICs (asterisks) after the E2 puff (scale bar: 5 pA, 500 ms). (**E**) SIC frequency in 1 min bins before and after 10 s application of E2 (time = 0 min). Females: n = 12 cells from n = 3 mice; Males: n = 17 cells from n = 4 mice. (**F**) **Left:** Mean SIC frequency before and after 10 s application of E2 in female (before: 7.3 ± 1.4 min^−1^; after: 12.3 ± 1.7 min^−1^) and male (before: 8.5 ± 1.1 min^−1^; after: 12.0 ± 1.6 min^−1^) mice. Two-tailed Student’s paired t-test. **Right:** Mean SIC amplitude before and after 10 s application of E2 in female (before: 11.7 ± 1.2 pA; after: 12 ± 1.2 pA) and male (before: 10.9 ± 0.8 pA; after: 10.2 ± 0.6 pA) mice. Wilcoxon Rank Sum Test (female) and Two-tailed Student’s paired t-test (male). (**G**) Scheme of the experimental approach: in the presence of the estrogen receptor (ER) antagonist ICI 182,780 (ICI; 1 µM), a recording pipette was placed in a hippocampal pyramidal neuron (blue), and a puffing pipette was located nearby. Astrocytes are depicted in green and glutamate in red. (**H**) **Left:** Representative SIC traces in the presence of the ER antagonist ICI before and after application of E2 (10 µM) in female mice. Note that there is no increase in SICs (asterisks) after the E2 puff (scale bar: 5 pA, 500 ms). **Right:** Representative SIC traces in the presence of the ER antagonist ICI before and after application of E2 in male mice. Note that there is no increase in SICs (asterisks) after the E2 puff (scale bar: 5 pA, 500 ms). (**I**) SIC frequency in 1 min bins before and after 10 s application of E2 (time = 0 min) in the presence of ICI. Females: n = 9 cells from n = 3 mice; Males: n = 11 cells from n = 3 mice. (**J**) **Left:** Mean SIC frequency in the presence of ICI before and after 10 s application of E2 in female (before: 8.6 ± 2.3 min^−1^; after: 8.6 ± 2.5 min^−1^) and male (before: 7.8 ± 1.1 min^−1^; after: 9.3 ± 1.7 min.^−1^) mice. Wilcoxon Rank Sum Test (female) and Two-tailed Student’s paired t-test (male). **Right:** Mean SIC amplitude in the presence of ICI before and after 10 s application of E2 in females (before: 11.1 ± 0.7 pA; after: 10.3 ± 0.9 pA) and males (before: 11.2 ± 0.5 pA; after: 10.6 ± 0.8) mice. Two-tailed Student’s paired t-test. Data are expressed as mean ± standard error of the mean (SEM), * *p* < 0.05; ** *p* < 0.005

Astrocyte calcium elevations are known to stimulate the release of neuroactive substances called gliotransmitters that can regulate neuronal activity and synaptic transmission [54]. In particular, astrocyte glutamate has been found to activate extrasynaptic NMDA receptors on neurons, which produce slow inward currents (SICs) [27, 39, 43, 55–57]. These SICs have a distinct characteristic of slower kinetics compared to excitatory post-synaptic currents and have been observed in multiple brain regions, including the hippocampus [28, 30, 44, 58] (Fig. 4C). In order to study the effects of E2 on astrocyte signaling, we conducted electrophysiological recordings of CA1 pyramidal neurons in the presence of tetrodotoxin (TTX) to block sodium-dependent action potentials and in the absence of extracellular Mg^2+^ to optimize the activation of NMDA receptors. After recording spontaneous SICs for 3 min, we locally applied E2 with the micropipette. We found that local application of E2 increased the frequency of SICs in both female and male mice (Fig. 4D-F), with no changes in SIC amplitude (Fig. 4F). To confirm that the effects of E2 were mediated by the activation of estrogen receptors, we conducted experiments in the presence of the ER antagonist ICI. We found that in the presence of ICI, E2 no longer induced an increase in SIC frequency (Fig. 4G-J), verifying that the E2-evoked increase in glutamate release by astrocytes requires estrogen receptor activation.

Taken together, these results indicate that E2 stimulates the release of the gliotransmitter glutamate from astrocytes.

### E2 Regulates Excitatory Synaptic Transmission Through Activation of Astrocytes in Female, but not Male, Mice

**Fig. 5:**
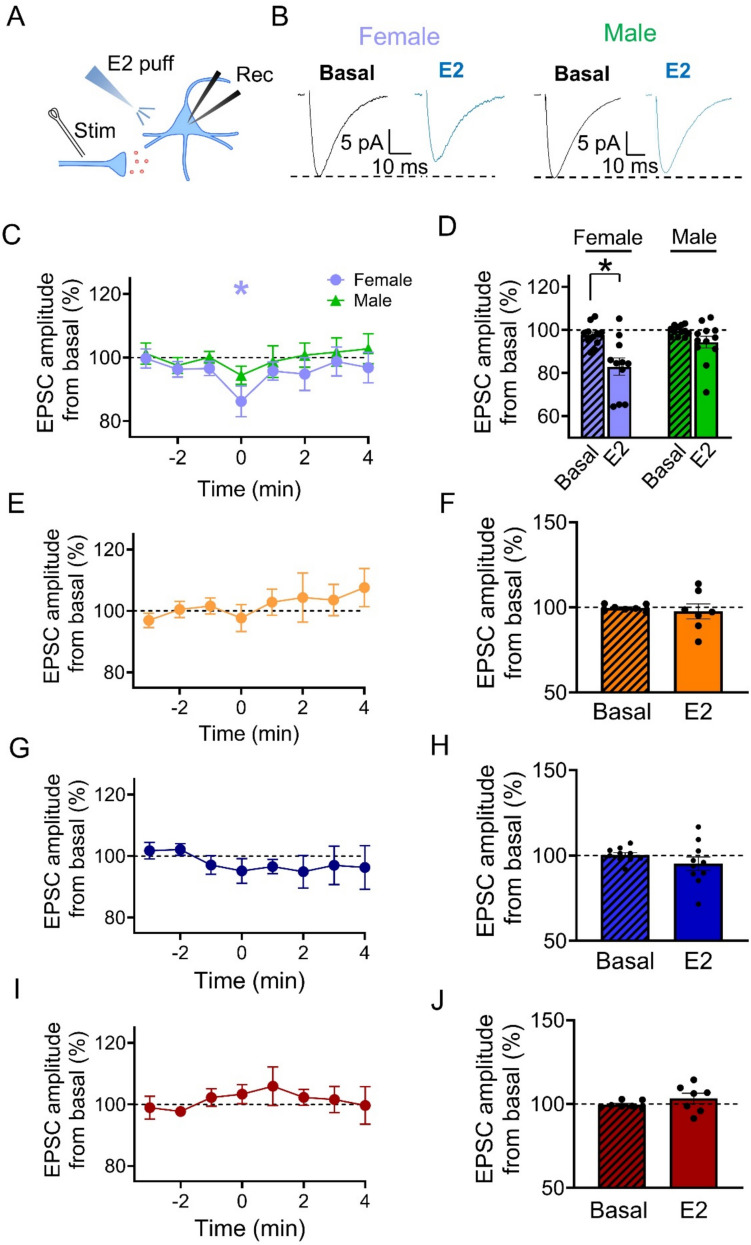
E2 regulates excitatory synaptic transmission in female mice. (**A**) Scheme of the experimental approach: a recording pipette was placed in a hippocampal pyramidal neuron (blue) and a puffing pipette was located nearby. An electric stimulator is placed in the *stratum radiatum* to stimulate the Schaffer collaterals. Glutamate is depicted in red. (**B**) **Left:** representative examples of excitatory postsynaptic currents (EPSCs) before and after application of E2 (10 µM) in female mice (scale bar: 5 pA, 10 ms). Note the decrease of EPSC amplitude after the E2 puff. **Right:** representative traces of EPSCs before and after application of E2 in male mice. (**C**) Normalized EPSC amplitude in 1 min bins before and after 10 s application of E2 (time = 0). (**D**) Mean EPSC amplitude before and after 10 s application of E2 in female (before: 97.5 ± 1.6%; after: 83 ± 4.0%) and male (before: 99.7 ± 0.7%; after: 94.4 ± 2.7%) mice. Females: n = 11 cells from n = 5 mice; Males: n = 12 cells from n = 3 mice. (**E**) Normalized EPSC amplitude in 1 min bins before and after 10 s application of E2 (time = 0) in the presence of ICI 182,780 (ICI; 1 µM) in female mice. (**F**) Mean EPSC amplitude before and after 10 s application of E2 in the presence of ICI in female mice (before: 99.7 ± 0.7%; after: 97.7 ± 4.4%). N = 7 cells from n = 2 mice. (**G**) Normalized EPSC amplitude in 1 min bins before and after 10 s application of E2 (time = 0) in conditional IP3R2^−/−^ female mice. N = 10 cells from n = 2 mice. (**H**) Mean EPSC amplitude before and after 10 s application of E2 in conditional IP3R2.^−/−^ female mice (before: 100.3 ± 1.3%; after: 95.2 ± 4.0%). (**I**) Normalized EPSC amplitude in 1 min bins before and after 10 s application of E2 (time = 0; asterisk) in the presence of CPT (10 µM) in female mice. N = 7 cells from n = 3 mice. (**J**) Mean EPSC amplitude before and after 10 s application of E2 in the presence of CPT in female mice (before: 99.7 ± 0.8%; after: 103.4 ± 3.1%). Two-tailed Student’s paired t-test. Data are expressed as mean ± standard error of the mean (SEM), * *p* < 0.05

Hippocampal astrocytes have been shown to regulate synaptic transmission and plasticity through the release of different gliotransmitters that activate neuronal receptors [28, 59–62]. These gliotransmitters include glutamate, which activates presynaptic mGluRs and potentiates synaptic transmission [30, 63]. D-serine acts as a co-agonist of postsynaptic NMDARs and contributes to long-term potentiation [64]. ATP, which is extracellularly converted to adenosine, acts on presynaptic A1 receptors and depresses neurotransmission [65–67]. We therefore investigated the consequences of the E2-induced astrocyte Ca^2+^ elevations on excitatory synaptic transmission in the hippocampus. We performed whole-cell patch clamp recordings from CA1 pyramidal neurons and evoked excitatory postsynaptic currents (EPSCs) by electrical stimulation of Schaffer collaterals. We monitored EPSCs before and after the local application of E2 (Fig. 5A). In female mice, E2 transiently depressed EPSC amplitude (from 97.5 ± 1.6% to 83 ± 4.0%) (Fig. 5B-D). In contrast, this effect was absent in male mice (from 99.7 ± 0.7% to 94.4 ± 2.7%) (Fig. 5B-D). Consistent with the effects on astrocyte Ca^2+^ and glutamate release, the synaptic effects were abolished by ICI (from 99.7 ± 0.7% to 97.7 ± 4.4%) (Figs. 5E,F). These results indicate that E2 acts via estrogen receptors to depress excitatory synaptic transmission in female mice. Next, we tested whether the astrocyte activity was necessary for the E2 modulation of excitatory synaptic transmission. We used conditional IP3R2^−/−^ mice, in which G protein-mediated Ca^2+^ elevations are largely impaired in astrocytes. In slices from these mice, astrocyte Ca^2+^ levels (Fig. 3D-F) and synaptic transmission (Fig. 5G,H) were both unaffected by E2 (from 100.3 ± 1.3% to 95.2 ± 4.0%), suggesting that the E2-evoked synaptic regulation requires astrocyte activation. Since astrocytes can depress excitatory synaptic transmission via gliotransmission of ATP/adenosine, we used CPT to block A1 receptors. We found that in the presence of CPT, synaptic transmission was unaffected (from 99.7 ± 0.8% to 103.4 ± 3.1%), indicating that E2-evoked synaptic transmission requires A1 receptor activation (Fig. 5I,J). Together, these results indicate that activation of G protein signaling in astrocytes is necessary for E2 depression of EPSCs in the hippocampus of female mice.

## Discussion

Present results show that astrocytes in the hippocampus express estrogen receptors and respond to local application of E2. This, in turn, induces an astrocyte calcium signal that was manifested as increases in the frequency of calcium events, i.e., transient intracellular calcium elevations. The effect of E2 on the astrocyte calcium signal was accompanied by an enhancement of the frequency of neuronal SICs, known to be mediated by activation of neuronal NMDARs following the release of the gliotransmitter glutamate [37–40]. Finally, E2 application in female mice elicited a transient depression of excitatory synaptic transmission that was sensitive to the antagonist of adenosine A1 receptors. The fact that the E2-evoked stimulation of the astrocyte calcium signal, the increase in the frequency of SICs, and the regulation of synaptic transmission were prevented by ICI and were absent in conditional IP3R2^−/−^ mice indicated that these phenomena were mediated by ER activation and required the IP3-mediated astrocyte calcium mobilization. Notably, while the E2-induced synaptic regulation was observed in female mice, we failed to detect significant changes in male mice, indicating that the neuromodulatory synaptic effects of E2 were sex-dependent. Overall, the data indicate astrocytes contribute to E2 signaling in the hippocampus.

Our results show that E2 increased the Ca^2+^ event frequency in astrocytes independently of the phase of the estrous cycle (see Fig. 2G). These effects were observed upon exogenous local application of E2 in hippocampal slices. This suggests that circulating E2 is not mediating these changes in Ca^2+^ event frequency since these effects occurred during fast, high concentrations of E2. This application could potentially model high concentrations of E2 that occur locally at the synapse. There were also differences in basal calcium activity across the estrous cycle. While outside the scope of this work, this is a phenomenon that awaits future study. Future studies in vivo, including ovariectomized female mice, are needed to further understand the role of E2 signaling in astrocytes in vivo.

Here, we show using in situ hybridization and immunofluorescence that both males and females express ERs in hippocampus CA1 astrocytes. The expression of ER by different neuronal populations has been widely studied [68–71]. While there has been some debate about the presence of ER in non-neuronal cells, their expression in glial cells has now been observed both in vitro and ex vivo. Indeed, various studies have reported the expression of both ERα and ERβ in astrocytes in cell culture [16, 72–75]. There are conflicting results about the expression pattern of ERs in astrocytes in the hippocampus as well as other brain regions. For instance, ERα immunoreactivity has been observed in hippocampal astrocytes of rats [25] as well as in the olfactory bulb and hypothalamus [22, 23]. ERβ expression has been found in astrocytic subpopulations in the adult rat brain [21, 24]. The reported discrepancies in experimental findings may arise from the well-known phenotypic changes of astrocytes in different culture conditions and the low level of ER expression in astrocytes, which could make its detection difficult, especially with the less sensitive techniques that were previously used. However, its expression appears to be increased under pathophysiological conditions. An increase in ERα expression has been shown to occur after brain injury in rats as well as in primates [76, 77]. On the other hand, ERα is increased in the hippocampus in patients with Alzheimer’s Disease [78]. Using RNAscope, here we show that hippocampal astrocytes in situ express both ERα and ERβ. Further studies, including analysis of ER protein levels and localization, are needed to understand the proportion of nuclear and membrane ERs in the hippocampal astrocytes.

Currently, two primary classes of ERs are known to exist, the traditional estrogen receptors ERα and ERβ, which are capable of being trafficked to the surface membrane to activate mGluR signaling, and the G protein-coupled receptor, GPER [1, 79]. In the present study, we observed that local application of E2 elicited relatively fast actions (within 10 s) on stimulating calcium signaling, gliotransmitter release, and modulating synaptic transmission. Therefore, it is likely that the observed results were mediated by the interaction of E2 with a membrane-bound receptor. That said, the mechanism by which this occurs does not fit a previously identified model. The effects appear dependent on ERα and/or ERβ (i.e. antagonism by ICI 182,780) but do not require mGluR activation.

This study adds to previous research on the effects of E2 hormone signaling on the function of astrocytes. Previous studies have shown that estrogen enhances the growth of astrocytic processes in the hypothalamus, which results in a decrease in the number of inhibitory synapses [80–83]. Additionally, estradiol increases the production of glial fibrillary acidic protein (GFAP) and supports the differentiation of astrocytes both in vitro and in vivo, in the hypothalamus and hippocampal formation [80, 84–86]. Furthermore, many studies have found that estrogen provides neuroprotection against neurodegenerative diseases and neuroinflammation [87–90]. One study concluded that ERβ on astrocytes in the hippocampus is neuroprotective against aging-related cognitive impairment [91]. It is yet to be investigated whether the effects observed involve an E2-mediated communication between astrocytes and neurons. However, the current findings suggest that astrocytes are responsive to E2 through activation of estrogen receptors, leading to an increase in the release of gliotransmitters and modulation of synaptic transmission. This indicates that there is a two-way communication system that is triggered by E2, which could have significant implications for brain physiology and pathophysiology.

While the present study used local exogenous application of E2 to investigate the astrocytic responsiveness to E2, the potential physiological source of E2 remains to be explored. The source of circulating E2 is predominantly from the gonads [10, 92], but estradiol can also be synthesized in the brain via local aromatase expression that converts testosterone into estradiol [88, 93]. Further studies are needed to clarify this issue, with the possibility that both gonadal-derived and brain-derived estradiol may both contribute to astrocyte activity.

Astrocyte calcium elevations are known to stimulate the release of gliotransmitters, such as glutamate, which can impact neuronal and synaptic activity [26]. Many studies have shown that astrocytic glutamate release generates SICs (slow inward currents) by activating extrasynaptic NMDA receptors that contain the GluN2B subunit. In the hippocampus and nucleus accumbens, enhanced neuronal synchronization or excitability has been observed following SICs [55, 94]. In this study, we found that E2 activates the astrocyte calcium signal and enhances the frequency of glutamate-mediated SICs, suggesting that E2 may influence local neuronal network activity through the activation of astrocytes. Our results also show that E2 regulates excitatory synaptic transmission in female mice, decreasing the EPSC amplitude (Fig. 5). This phenomenon was found to be mediated by the astrocyte calcium signal because it was absent in conditional IP3R2^−/−^ mice, where calcium signaling is impaired in astrocytes [44, 52, 53]. These results present some discrepancies with previous findings that showed an increase in glutamatergic synaptic transmission in the hippocampus of both male and female rats upon application of E2 [95]. Firstly, the different species used in the studies (rats vs mice) and the time frame of the E2 application might account for these differences. Moreover, in the present study, we observed fast and transient effects of E2 (within 10 s), while it took up to 10 min of E2 perfusion to observe the increase in the synaptic transmission shown in the Oberlander and Woolley study [95]. It is also worth mentioning that we failed to observe synaptic transmission modulation in male mice in the present study. These differences could be due to the differential expression of ERs between the sexes. Since astrocytes from female mice have more ER mRNA compared to male mice (Fig. 1), the subtle differences in EPSC amplitude observed in female mice might not be apparent in males. A longer exposure to E2 might reveal these alterations in synaptic transmission for male mice. Finally, while the effects described in rats were interpreted to be mediated by purely neuronal mechanisms, the E2-induced synaptic regulatory effects observed in this study are dependent on astrocyte calcium activity. Aside from ER expression, the fact that the E2-evoked calcium signal is similar in both sexes suggests that the sex-dependent differences in synaptic transmission most likely result from differences in the downstream processes of the calcium signal. Further studies are needed to examine possible differences in downstream calcium that lead to gliotransmitter release.

In the present study, E2, which was locally applied from a micropipette located approximately 50 microns over the stratum oriens of the hippocampal slice, could activate both neuronal and astrocyte estrogen receptors. However, several lines of evidence suggest that the reported effects of acute application of E2 are mediated by direct astrocyte activation. First, in calcium imaging experiments, the astrocyte calcium activity was isolated by preventing, or at least minimizing, the potential contribution of E2-induced neuronal activity using a cocktail of antagonists of neurotransmitter receptors along with TTX. Second, SIC recordings were performed in the presence of TTX. Finally, the fact that the effects of E2 on EPSCs were absent in conditional IP3R2^−/−^ mice strongly suggest that the neuronal contribution is, at least, negligible.

In summary, the current results demonstrate that E2 signaling impacts astrocyte-neuron communication in the hippocampus, identifying astrocytes as cellular elements involved in the physiology of estradiol signaling. Furthermore, considering the importance of estrogen signaling in numerous processes, such as reproduction and sexual differentiation or mood and cognition, present findings indicate that astrocytes may be actively involved in relevant phenomena of brain physiology.

## Materials and Methods

### Ethics Statement

All procedures for animals were approved by the Institutional Animal Care and Use Committee at the University of Minnesota and in compliance with the National Institutes of Health guidelines for the care and use of laboratory animals.

### Animals

C57BL/6 J female and male mice as well as IP3R2^−/−^ mice [96] or IP3R2 flox mice generously provided by Anna Dunaevsky (University of Nebraska Medical Center) were bred in-house with a 14-h light/10-h dark cycle. Conditional IP3R2^−/−^ mice were generated by stereotaxic injection of the virus AAV8-GFAP-CRE-mCherry in the hippocampus of IP3R2 flox mice, which allowed the selective deletion of IP3R2 in hippocampal astrocytes because the AAV-GFAP-Cre has robust expression in the hippocampus [97]. The vivarium was maintained at a controlled temperature of 22 °C and food and water were available ad libitum. All animals were at least 2 months old before experimental procedures. Vaginal smear cytology [92] was used to determine estrous cycle phase postmortem.

### Histology

Subjects were euthanized with a lethal dose of isoflurane (Piramal Critical Care, Bethlehem, PA) and sacrificed by rapid decapitation. Brains were extracted, frozen in optimum cutting temperature compound (VWR Scientific Products), and stored at − 80 °C. Brains were later sliced coronally on a cryostat at 14 µm thickness and sections were mounted on Colorfrost Plus slides (Fisher Scientific, Waltham, MA, USA). Slides were stored at − 80 °C until RNAscope procedure treatment.

### RNAscope with Immunofluorescence

The manufacturer’s protocol from the Multiplex fluorescent v2 assay produced by Advanced Cell Diagnostics (ACD) (Neward, CA, USA) was used.

Slides were fixed in 10% neutral buffered formalin for 15 min at room temperature. Slides were then rinsed twice with 1X phosphate-buffered saline (PBS). Afterward, the slides were dehydrated with increasing ethanol concentrations (50, 70, 100, 100%) at room temperature. Following dehydration, a hydrophobic barrier was drawn around each section on the slide. Sections were treated with 75 µL of Protease IV, left to incubate for 30 min, then washed twice with 1X PBS. ACD created two probes targeting ERα and ERβ in mice: Mm-Esr1-C2 (mRNA encoding Estrogen Receptor α); GenBank accession number (NM_007956.5, target nt region 678–1723) and Mm-Esr2-C3 (mRNA encoding Estrogen Receptor β); GenBank accession number (NM_207707, target nt region 424–1875). Each probe mixture contained a 50:1 ratio of probe diluent and C2/C3. After applying 75 µL of probes to each slice, the slides were incubated at 40 °C for 2 h. After incubation, the slides were washed twice with washing buffer (WB; 1X SSC + SDS; 8.7 g/l of sodium chloride, 4.41 g/l of sodium citrate, and 3 g/l of sodium dodecyl sulfate). Each slide was incubated with amplifying reagents (AMP1, AMP2, AMP3) at 40 °C for 30 min, 30 min, and 15 min, respectively, with two 2-min washes with WB in between each AMP treatment. Following this, the slides then underwent incubation in horseradish peroxidase (HRP) corresponding with each channel for 15 min at 40 °C with two 2-min washes in WB afterward. Sections were then treated with 75 µL Opal dye (Akoya Biosciences, Marlborough, MA, USA) and incubated for 30 min at 40 °C. All Opal dyes were diluted to a concentration of 1:750 using TSA buffer (Advanced Cell Diagnostics). Fluorescently labeled probes were applied so that ERα-C2 was labeled with Opal 570 and ERβ-C3 with Opal 650. Slides were then washed twice in WB for 2 min. Sections were treated with an HRP-blocker and incubated for 15 min at 40 °C.

For immunofluorescence, the slides were washed and then incubated with 100 µL of primary antibody solution on each slide (GFAP anti-mouse from Cell Signaling, Beverly, MA, USA) in 1:1000 dilution in 1X Tris-buffered saline (TBS) + 0.25% Gelatin + 0.5% Triton-X overnight at 4 °C. After incubation, the primary antibody was washed 3 times with TBS for 10 min each. Then the slides were incubated with secondary antibody solution (488 Goat anti-mouse from Invitrogen) at 1:400 dilution in 1XTBS + 0.25% Gelatin + 0.5% Triton-X for 1 h at room temperature. After incubation, the slides were washed with TBS 3X for 10 min. After air drying, Prolong Gold antifade reagent with DAPI was applied to each slide and cover slipped. All slides were stored at 4 °C at least overnight before imaging.

### Confocal Imaging

All imaging for RNAscope was performed using Olympus BX2 Upright Confocal provided by the University of Minnesota Imaging Center. Images were collected from both the left and right hemispheres of each slice. With 4 slides per animal, there were approximately 8 images for each animal. Images were collected at 20X/0.60 objective at 1,024 X 1,024-pixel distribution. Each z-stack had 1.5 µm spacing using 488, 549, and 647 nm lasers.

### Image Analysis

All images were analyzed using Imaris software (Oxford Instruments, version 9.7.2). Each image was a composite of DAPI (blue), GFAP (green), ERα (red), and ERβ (magenta). Each image was analyzed using either the surface or spots model.

#### Surface Model

For DAPI, the seed point diameter was set to 5 µm. Afterward, three filters were applied: Quality, Area, and Volume to include as many cells as possible. For GFAP, the surface detail was set to 1.24 µm and the same filters were applied.

#### Spots Model

For both ERα and ERβ, the spots model was used to quantify puncta of mRNA. For both channels, the estimated XY diameter was set to 0.750 µm. Then the quality filter was applied to include as many spots as possible. Then the “average distance to 3 nearest neighbors” filter was applied for all spots that passed the quality filter.

#### Colocalization

To colocalize puncta of ERα and ERβ mRNA with DAPI and GFAP, a mask channel was created in Imaris. The GFAP surface model was masked onto the DAPI surface model to create a Masked DAPI channel. After running the Masked DAPI channel through the surfaces model and applying the same three filters, the"Find Spots Close to Surface"function was used to determine which spots were within 1 µm of the Masked DAPI surfaces. To colocalize the puncta of mRNA with GFAP only, the same"Find Spots Close to Surface"parameters were used.

### Stereotaxic Surgery

Female and male mice were anesthetized with isoflurane and given a bilateral injection (1µL) of AAV5-GfapABC1D-cytoGCaMP6f (Addgene, MA, USA; cat #: 52925-AAV5) (titer: 2.2 × 10^13^ gc/mL) or AAV8-GFAP-Cre-mCherry (titer: 3.8 × 10^12^ virus molecules/mL) (Viral Vector and Cloning Core, University of Minnesota, USA) in the dorsal CA1 hippocampus (AP: − 1.82 mm; ML: ± 1.30 mm; DV: − 1.20 mm). The injection was given via a Hamilton syringe with a 29-gauge needle at a rate of 0.8–1.25 µl/minute. Ketoprofen was given post-operatively for 3 days at 5 mg/kg.

### Dorsal CA1 Hippocampus Slice Preparation

Mice were rapidly decapitated, and the brain was quickly extracted. The brain was placed in an ice-cold sucrose solution (sucrose 189 mM, glucose 10 mM, NaHCO_3_ 26 mM, KCl 3 mM, MgSO_4_ 5 mM, CaCl_2_ 0.1 mM, NaH_2_PO_4_ 1.25 mM). Brains were sliced at 350 µm thick using a vibratome (Leica VT12005). All brain slices were placed in oxygenated (95% O_2_/5% CO_2_) artificial cerebrospinal fluid (ACSF) (NaCl 124 mM, KCl 2.69 mM, KH_2_PO_4_ 1.25 mM, MgSO_4_ 2 mM, NaHCO_3_ 26 mM, CaCl_2_ 2 mM, and glucose 10 mM solution) (pH = 7.3–7.4) and recovered for a minimum of 30 min before imaging or electrophysiological experiments. Slices were placed in a recording chamber and superfused (2 mL/min) with oxygenated ACSF. Olympus BX51 WI microscope confocal microscope (Olympus Optical, Tokyo, Japan) equipped with a ThorLabs camera was used for the experiments.

### Calcium Imaging

After stereotaxic surgery, mice were left to recover for at least 3 weeks to obtain viral optimal expression. Mice were sacrificed via rapid decapitation and brains were immediately placed in iced sucrose cutting solution. For imaging, slices were perfused with ACSF containing TTX (1 µM) to block voltage-gated sodium channels, CNQX (20 µM) to block AMPA receptors, AP5 (50 µM) to block NMDA receptors, MPEP (50 µM) to block metabolic glutamate 5 receptors, LY367385 (100 µM) to block metabotropic glutamate 1a receptors, picrotoxin (50 µM) to block GABA_A_ receptors, CGP5462 (1 µM) to block GABA_B_ receptors, atropine (50 µM) to block muscarinic receptors, CPT (8.8 µM) for adenosine A1 receptors, AM251 (2 µM) to block endocannabinoid receptors, flupentixol (30 µM) to block dopamine (D1, D2, D3 and D5) receptors and suramin (100 µM) to block purinergic receptors. The same control solution with the estrogen receptor antagonist ICI (1 µM) was used as well. All images were recorded at 1 frame/second. 17β-estradiol (E2) (MilliporeSigma, Germany) was prepared for pressure pulse application at 10 µM. The pressure pulse was applied at 20 psi for 10 s from a micropipette located approximately 50 microns over the stratum oriens. Stratum oriens, stratum pyramidal and stratum radiatum of CA1 were present in the field of view, and activity in all three regions was measured.

All recordings were analyzed using AQuA software using MATLAB (MathWorks, Natick, MA, USA). We used automatic event detection in AQuA as detailed in [98] to quantify activity in the soma. All videos from C57BL/6 J mice were analyzed using the following settings: intensity threshold scaling factor 1, smoothing (sigma) 0.5, minimum Size (pixels) 20, temporal cut threshold 2, growing z threshold 1, rising time uncertainty 2, slowest delay in propagation 2, propagation smoothness 1, z score threshold 2, maximum distance 0, minimum correlation 0, maximum time difference 2, temporally extended events disabled, ignore delay tau not enabled. For proofreading, events had to be a minimum of 0.5 ΔF/F and 3 s duration. All videos from IP3R2^−/−^ mice were analyzed using the following settings: intensity threshold scaling factor 1, smoothing (sigma) 0.5, minimum Size (pixels) 30, temporal cut threshold 2, growing z threshold 1, rising time uncertainty 2, slowest delay in propagation 2, propagation smoothness 1, z score threshold 2, maximum distance 0, minimum correlation 0, maximum time difference 2, temporally extended events disabled, ignore delay tau not enabled. For proofreading events, only events with a minimum of 0.5 ΔF/F and a 3-s duration were considered. The frequency of the calcium events was the number of events detected over a 10 s bin. For calcium imaging experiments, all data were normalized to 50 s before the E2 application.

### Electrophysiology

Whole-cell patch clamp electrophysiology recordings were conducted in CA1 pyramidal neurons of the dorsal hippocampus. Slow inward currents (SICs) were recorded in a Mg^2+^ free ACSF solution (NaCl 124 mM, KCl 2.69 mM, KH_2_PO_4_ 1.25 mM, NaHCO_3_ 26 mM, CaCl_2_ 4 mM, glucose 10 mM, and glycine 10 μM) with 1 µM tetrodotoxin (TTX). Magnesium was not included in the solution to optimize the activation of NMDA receptors. Experiments were performed with the temperature of the bath solution kept at 34ºC with a temperature controller TC- 324B (Warner Instruments Co.). The internal solution in the pipette consisted of KMeSO_4_ 135 mM, KCl 10 mM, HEPES 10 mM, NaCl 5 mM, ATP–Mg^2+^ 2.5 mM, and GTP–Na^+^ 0.3 mM (pH = 7.3) with an electrode resistance between 3 and 10 MΩ. The membrane potential of neurons was held at − 70 mV. Series and input resistances were monitored throughout the experiment using a − 5 mV pulse. A baseline was recorded for 3 min. Then, E2 was applied via pressure pulse for 10 s and SICs were recorded for 5 min. To record evoked excitatory postsynaptic currents (EPSCs), theta capillaries filled with ACSF were used for bipolar stimulation and placed in the *stratum radiatum* to stimulate the Schaffer collaterals. Electrical pulses were continuously delivered at 0.33 Hz. EPSCs were isolated using picrotoxin (50 µM) and CGP5462 (1 µM) to block GABA_A_ and GABA_B_ receptors, respectively. Since astrocytes can depress excitatory synaptic transmission via gliotransmission of ATP/adenosine, we used CPT (10 µM) to block A1 receptors. After a stable baseline of several minutes was obtained, E2 was applied for 10 s and EPSCs were recorded for several minutes. The amplitudes of EPSCs were grouped in 1-min time bins. The baseline mean EPSC amplitude was obtained by averaging the mean values obtained within 3 min of baseline recordings. The mean EPSC amplitudes were then normalized to the baseline. To assess changes in EPSC amplitude, the effect of E2 was statistically tested by comparing the normalized EPSCs recorded 3 min before and 1 min after the stimulus. Signals were filtered at 1 kHz and acquired at 10 kHz sampling rate. pCLAMP 10.4 (Molecular Devices, Sunnyvale, CA, USA) was used for data collection and analysis. SICs were distinguished from mEPSCs by their significantly slower kinetics, as previously described [30, 58]. Briefly, SICs were defined as currents with a rise time greater than 5 ms, and a decay time exceeding 10 ms.

### Statistical Analysis

Data normality was tested using a Shapiro–Wilk test. All data in the figures were analyzed using Student’s paired and unpaired t-tests unless the data was not normally distributed. Then either the Wilcoxon Test (α = 0.05) or Mann–Whitney U test was used. All data are expressed as mean ± standard error of the mean (SEM). Statistical significance was defined as a p-value less than 0.05 (*), 0.005 (**). All analysis was performed using GraphPad 10.1 (San Diego, CA, USA).

## Data Availability

All data from the figures is included in the manuscript. Data is available upon request from the author.
